# Three-dimensional transesophageal echocardiographic morphological evaluation of the tricuspid valve

**DOI:** 10.1093/icvts/ivac145

**Published:** 2022-05-30

**Authors:** Takumi Kawase, Yosuke Takahashi, Asahiro Ito, Hisako Yoshida, Yosuke Sumii, Kenta Nishiya, Noriaki Kishimoto, Kokoro Yamane, Yoshito Sakon, Akimasa Morisaki, Hiromichi Fujii, Toshihiko Shibata

**Affiliations:** Department of Cardiovascular Surgery, Osaka City University Graduate School of Medicine, Osaka, Japan; Department of Cardiovascular Surgery, Osaka City University Graduate School of Medicine, Osaka, Japan; Department of Cardiology, Osaka City University Graduate School of Medicine, Osaka, Japan; Department of Medical Statics, Osaka City University Graduate School of Medicine, Osaka, Japan; Department of Cardiovascular Surgery, Osaka City University Graduate School of Medicine, Osaka, Japan; Department of Cardiovascular Surgery, Osaka City University Graduate School of Medicine, Osaka, Japan; Department of Cardiovascular Surgery, Osaka City University Graduate School of Medicine, Osaka, Japan; Department of Cardiovascular Surgery, Osaka City University Graduate School of Medicine, Osaka, Japan; Department of Cardiovascular Surgery, Osaka City University Graduate School of Medicine, Osaka, Japan; Department of Cardiovascular Surgery, Osaka City University Graduate School of Medicine, Osaka, Japan; Department of Cardiovascular Surgery, Osaka City University Graduate School of Medicine, Osaka, Japan; Department of Cardiovascular Surgery, Osaka City University Graduate School of Medicine, Osaka, Japan

**Keywords:** Tricuspid valve, Two posterior leaflets, Three-dimensional transesophageal echocardiography

## Abstract

**OBJECTIVES:**

The morphology of the tricuspid valve (TV), particularly valves with two posterior leaflets, is attracting attention. The present study was performed to investigate the usefulness of three-dimensional transoesophageal echocardiographic data for morphological evaluation of the TV .

**METHODS:**

Sixty patients underwent morphological evaluation of the TV by preoperative transoesophageal echocardiography followed by TV repair with median sternotomy, and each leaflet was measured intraoperatively. We analysed the TV morphology in 51 patients whose preoperative echocardiographic findings were consistent with intraoperative findings.

**RESULTS:**

The mid-systolic echo data, which included the annulus diameter of each leaflet, were correlated with the intraoperative evaluation findings compared with those in the mid-diastole. The annulus and area of the posterior leaflet were larger in patients with two than one posterior leaflet valve (42.4 ± 13.5 vs 30.7 ± 9.1 mm, *P* < 0.001 and 327 ± 185 vs 208 ± 77 mm^2^, *P* = 0.006, respectively). In the severe tricuspid regurgitation patients, the annulus of the posterior leaflet was larger and the annulus of the anterior leaflet was smaller in patients with two than one posterior leaflet valve [posterior: 48 mm [95% confidence interval (CI), 41–54 mm] vs 36 mm (95% CI, 27–45 mm), respectively; *P* = 0.043 and anterior: 38 mm (95% CI, 33–42 mm) vs 46 mm (95% CI, 40–52 mm), respectively; *P* = 0.025].

**CONCLUSIONS:**

Patients who had a TV with two posterior leaflets had a larger annulus and area of the posterior leaflets. Preoperative three-dimensional transoesophageal echocardiography is useful for the morphological evaluation of the TV.

**Figure:**
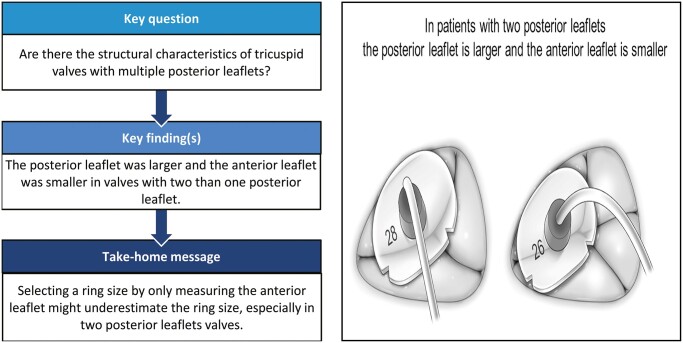

## INTRODUCTION

Tricuspid valve (TV) repair is often performed as a concomitant procedure of left cardiac surgery. Recently, following the establishment of the 2020 Japan JCS/JSCS/JATS/JSVS Guideline, the indications for surgical treatment of TV expanded to include mild or moderate tricuspid regurgitation (TR) in patients with tricuspid annular dilation or atrial fibrillation [1]. As TR treatment has become more widespread, the mechanism of TR and the anatomy of the TV have become growing concerns that are attracting increasing attention.

Regarding the morphological diversity of the TV, Silver *et al.* [2] reported that the posterior leaflet of the TV actually consists of several leaflets. In addition, Sakon *et al.* [3] reported that half of the autopsied hearts they examined had TVs with two or more posterior leaflets and that such valves showed anatomical differences in annular length. The TV annulus is a three-dimensional (3D) configuration with a saddle shape. However, as the TV annulus expands, the 3D structure is lost and becomes flattened [4]. In addition, enlargement of the annulus is greater on the free-wall side, and the morphology of the TV changes depending on the degree of TR [5]. Therefore, preoperative evaluation of the TV morphology in patients with TR is important to improve the surgical outcome.

Recent advances in 3D echocardiographic technology have made it possible to evaluate the TV morphology using 3D imaging. Some previous studies of 3D transthoracic echocardiography or 3D transoesophageal echocardiography (TEE) indicated that preoperative 3D images were useful to assess predictors of residual TR. However, these reports only evaluated leaflet parameters such as tethering, the total area and the annulus [6, 7]; no reports have focused on morphological aspects such as multiple posterior leaflets.

In the present study, we compared preoperative 3D image evaluation of the TV with intraoperative measurement of the TV and evaluated the accuracy of preoperative 3D TEE imaging. We also evaluated the structural characteristics of TVs with two posterior leaflets and their impact on TR severity.

## MATERIALS AND METHODS

### Ethics statement

This study was approved by the Osaka City University Ethical Review Board (approval no. 2020-099). Informed consent was obtained from all patients using an opt-out method.

### Patients

From April 2016 to November 2020, 60 patients were diagnosed with secondary TR associated with left heart disease and underwent TV repair with median sternotomy. All patients underwent preoperative TEE and measurement of the diameter of the TV annulus. The exclusion criteria were infective endocarditis and primary TR. Patients without perioperative echocardiographic data and patients who underwent re-do TV repair were also excluded.

Among the 60 patients, we collected data from 51 patients in whom the number of TV leaflets was correctly determined by preoperative TEE in comparison with intraoperative findings. We performed the following 3 analyses. (i) We compared the annulus diameter and the number of TV leaflets as determined by preoperative 3D TEE versus intraoperative measurement. In addition, we examined which of the 2 time phases (mid-systole or mid-diastole) of the measured parameters approximated the intraoperative measurement result. (ii) We compared the echocardiographic parameters of TVs with one versus two posterior leaflets. (iii) To determine whether a relationship was present between the TR grade and the degree of TV annular enlargement in patients who had a TV with one versus two posterior leaflets, we classified the patients into those with ≤mild, moderate or severe TR and compared the size of each leaflet among the groups.

### Transthoracic echocardiography

All patients underwent preoperative transthoracic echocardiography using an iE33 or Epiq system (Philips Medical Systems, Andover, MA, USA) at our echocardiography laboratory. The TR grade was defined using a multiparametric approach, including an assessment of the colour Doppler-derived jet area, the continuous-wave Doppler-derived jet density and contour and the hepatic vein flow velocity pattern [8]. Continuous-wave Doppler was used to obtain the TR peak velocity (m/s) and the transtricuspid systolic pressure gradient (mmHg), which was calculated as 4*V*^2^ (where *V* is velocity). The right ventricular systolic pressure was then estimated as the sum of the estimated transtricuspid systolic pressure gradient and right atrial (RA) pressure. An estimated right ventricular systolic pressure of >40 mmHg was considered indicative of pulmonary hypertension [9]. The tricuspid annular diameter was measured at end-diastole, and an annular diameter of >40 mm or 21 mm/m^2^ was considered significant annulus dilatation [1]. The RA dimension, left ventricular (LV) end-diastolic dimension, LV end-systolic dimension, LV ejection fraction and left atrial dimension were measured.

### Real-time three-dimensional transesophageal echocardiography

All patients underwent preoperative real-time 3D TEE using an iE33 or Epiq system (Philips Medical Systems) at our echocardiography laboratory.

The annulus length of each leaflet was determined by measuring the distance of the tracing length. The area of each leaflet was determined by measuring the tracing area.

Horizontal and vertical diameters were obtained by the QLAB mitral valve navigation (MVN)/3D quantification software package (Philips Medical Systems).

Determination of the commissure area and measurement of all parameters were supervised by a cardiologist of our institution. The number of TV leaflets was determined by identifying each commissure of the TV in the mid-systolic phase and mid-diastolic phase using 3D TEE with the centre of the ventricular septum at the 6 o’clock position.

**Figure 1:**
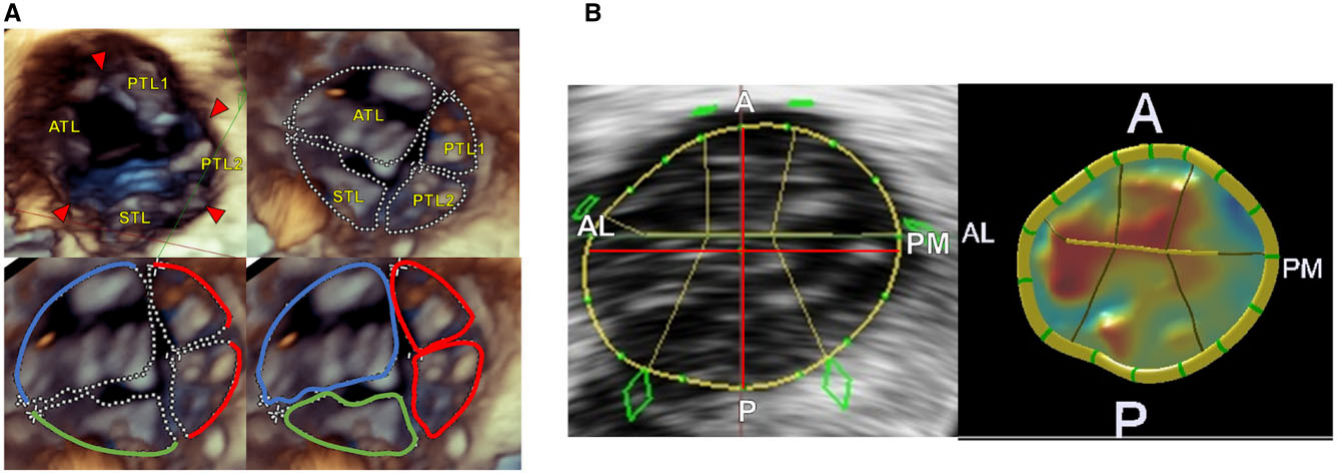
Determination of the number of leaflets and measurement of leaflets using three-dimensional images. (**A**) Tracing and measurement of each leaflet. (**B**) The anterior–posterior and anterolateral–posteromedial distances were determined by mitral valve navigation software. The arrowheads indicate the commissure. A: anterior; AL: anterolateral; ATL: anterior leaflet; P: posterior; PM: posteromedial; PTL1 and PTL2: posterior leaflets; STL: septal leaflet.

- We defined an indentation between two leaflets as a commissure. Each leaflet annulus was traced and measured in both phases. The annulus length of each leaflet was determined by measuring the length of the tracing line. To determine the leaflet area, each leaflet was traced and measured in mid-systole (Fig. 1A).
- In the quantification analysis using MVN, the line passing through the centre of the TV with reference to the intersection of the anterior and septal leaflets was defined as anterolateral-posteromedial, and the line perpendicular to this was defined as anteroposterior. We measured the anteroposterior and anterolateral-posteromedial distance, total area of the leaflets and total annulus diameter of the leaflets in both phases (Fig. 1B).

### Determination of the number of leaflets during operation

**Figure 2:**
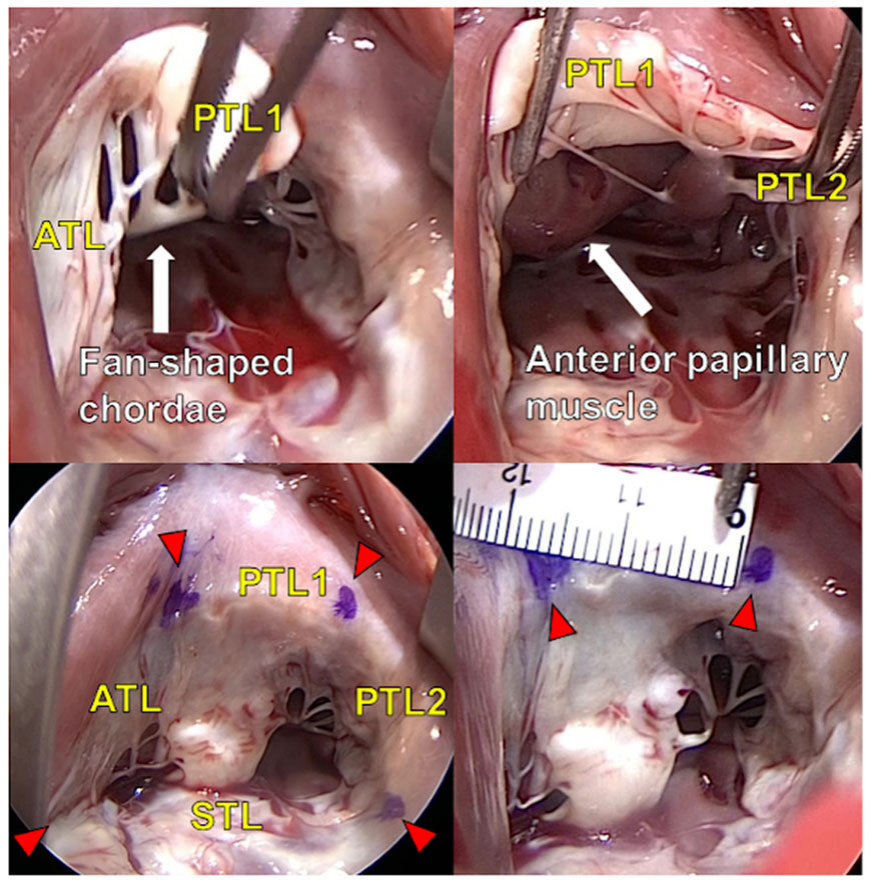
Operative findings of tricuspid valve with two posterior leaflets. The arrowheads indicate the commissure. ATL: anterior leaflet; PTL1 and PTL2: posterior leaflets; STL: septal leaflet.

The number of leaflets was determined according to the definitions presented by Silver *et al.* [2]: (i) the commissure was defined as indentation of the leaflets by a fan-shaped chorda, (ii) the fan-shaped chorda forming the anteroposterior commissure arose from the anterior papillary muscle and (iii) the posteroseptal commissure was defined by the fan-shaped chorda arising from the most medially located papillary muscle on the posterior wall (Fig. 2).

### Measurement between each commissure area during operation

The number of leaflets was confirmed, and each commissure was marked by pyoktanin blue (Sigma-Aldrich, St Louis, MO, USA). The distance between each parameter of the TV was measured directly using a paper measure (Fig. 2). All intraoperative evaluations were performed by the same surgical team.

### Statistical analysis

Descriptive statistics for categorical variables are reported as absolute value and percentage, and continuous variables are shown as mean and standard deviation. Categorical data were compared using the *X*^2^ test. Continuous variables were compared using the Wilcoxon signed-rank test. A regression model was performed to predict the annulus length and included an interaction of the TR level and the number of leaflets. All analyses were conducted with JMP version 13.0 (SAS Institute Inc., Cary, NC, USA), and a *P*-value of <0.05 was considered statistically significant.

## RESULTS

### Patients’ background

The patients’ profiles are shown in Table 1. Most of the patients (75%) had chronic or paroxysmal atrial fibrillation. Thirty-one patients (52%) had hypertension as a comorbidity. All patients had a history of congestive heart failure, and 22 patients (37%) had a history of hospitalization because of heart failure before surgery.

**Table 1:** Patient characteristics

Preoperative characteristics	*N* = 60	%
Age, years	72.6 ± 8.3	
Male sex	27	45
Body surface area, m^2^	1.5 ± 0.2	
New York Heart Association class		
I	8	13
II	42	70
III	8	13
IV	2	3
Admission due to heart failure	22	37
Comorbidities		
Atrial fibrillation	45	75
Hypertension	31	52
Dyslipidaemia	11	18
Diabetes	3	5
Ischaemic heart disease	6	10
Chronic obstructive pulmonary disease	4	7
Chronic renal failure	14	23
Cerebral infarction	8	13

Data are presented as *n* (%) or mean ± standard deviation.

### Preoperative echocardiographic data

The preoperative echocardiographic data are shown in Table 2. Eighteen patients (30%) had severe TR. Almost all patients (97%) underwent concomitant TV repair because of left heart disease, and 29 patients (48%) had pulmonary hypertension.

**Table 2:** Preoperative transthoracic echocardiographic data

19 April 2016 to 25 November 2020	*N* = 60	%
TR grade		
≤Mild	10	17
Moderate	32	52
Severe	18	30
Secondary TR due to left-sided valve disease	58	97
TRPG, mmHg	32 ± 12	
PH (estimated RVSP of >40 mmHg)	29	48
LVEF, %	58.0 ± 9.4	
LVDd (parasternal long-axis view), mm	51.5 ± 8.6	
LVDs (parasternal long-axis view), mm	32.0 ± 8.9	
LA minimum (parasternal long-axis view), mm	56 ± 12	
LA maximum (parasternal long-axis view), mm	74 ± 13	
RA minimum (four-chamber view), mm	43 ± 14	
RA maximum (four-chamber view), mm	62 ± 14	
TV diameter, mm/m^2^	24.8 ± 4.3	

Data are presented as *n* (%) or mean ± standard deviation.

LA: left atrium; LVDd: left ventricular diastolic dimension; LVDs: left ventricular systolic dimension; LVEF: left ventricular ejection fraction; PH: pulmonary hypertension (estimated right ventricular systolic pressure (RVSP) >40 mmHg); RA: right atrium; TR: tricuspid regurgitation; TRPG: transtricuspid pressure gradient; TV: tricuspid valve.

### Comparison of three-dimensional transesophageal echocardiography data with intraoperative measurements

Intraoperative examination showed that 31 patients (52%) had one posterior leaflet valve and 29 patients (48%) had two posterior leaflet valves.

The number of leaflets measured intraoperatively was equal to that as determined by preoperative 3D TEE in 51 of 60 patients (85%). The number of posterior leaflets in 9 patients (15%) did not match the TEE findings.

A comparison of the echo parameters between the 51 patients with correct measurements and the 9 patients with incorrect measurements showed that it was difficult to determine the number of leaflets in patients with a smaller RA diameter (minimum: 44.2 ± 13.9 vs 35.5 ± 9.9 mm, *P* = 0.017; maximum: 63.8 ± 14.7 vs 54.3 ± 9.7 mm, *P* = 0.033).

**Figure 3:**
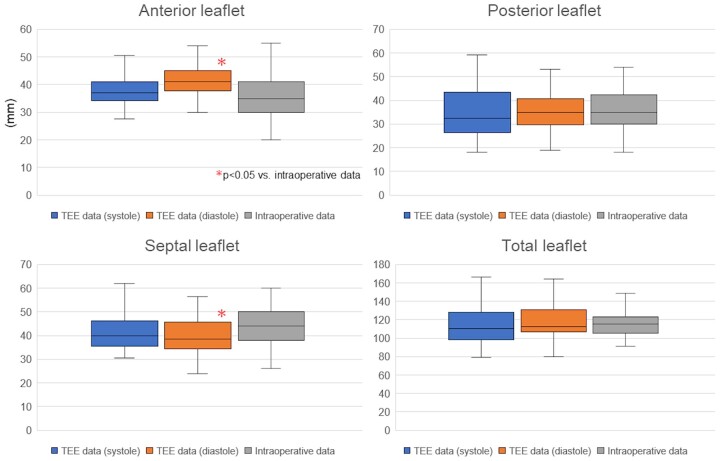
Comparison of transoesophageal echocardiography data and intraoperative parameters in the systolic or diastolic phase. TEE: transoesophageal echocardiography.

We investigated the 3D TEE parameters of the 51 patients in whom the number of leaflets was correctly identified by preoperative TEE. The whole annulus length and each annulus length were measured in both mid-systole and mid-diastole, and we compared the 3D TEE parameters of the 2 phases with the intraoperative parameters. There was no significant difference in the whole annular length and each annular length between the mid-systole data and intraoperative data. The annulus length of the anterior leaflet (TEE: 42.3 ± 9.7 mm vs intraoperation: 36.6 ± 9.6 mm, *P* < 0.001) and septal leaflet (TEE: 39.9 ± 7.8 mm vs intraoperation: 43.9 ± 7.6 mm, *P* = 0.003) showed significant differences, although there was no significant difference in the whole annulus length in the mid-diastole (Fig. 3).

### Comparison of parameters of tricuspid valves with 1 versus 2 posterior leaflets

**Figure 4:**
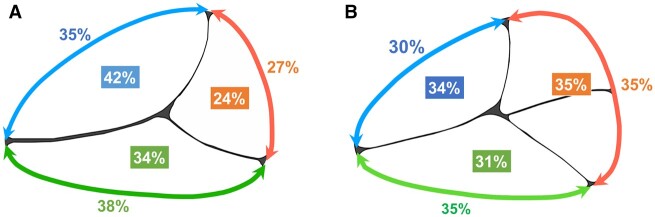
Percentage of annulus diameter and area of each leaflet. (**A**) Valve with one posterior leaflet. (**B**) Valve with two posterior leaflets.

The mid-systolic data, which did not differ from the intraoperative measurements, were used to compare the long and short diameters, the annulus length of each leaflet and the area of each leaflet between patients with one or two posterior leaflets (Table 3 and Fig. 4).

**Table 3:** Comparison of parameters of tricuspid valves with one versus two posterior leaflets

	Tricuspid valve with one posterior leaflet (*n* = 28)	Tricuspid valve with two posterior leaflets (*n* = 23)	*P*-Value
Severe TR	6 (21)	11 (48)	0.046
TRPG, mmHg	29.0 ± 8.7	33.8 ± 11.9	0.166
PH (estimated RVSP of >40 mmHg)	10 (36)	15 (65)	0.034
LVEF, %	57.6 ± 9.7	59.1 ± 8.8	0.543
LVDd (parasternal long-axis view), mm	50.6 ± 8.3	51.7 ± 9.4	0.910
LVDs (parasternal long-axis view), mm	30.8 ± 7.2	32.6 ± 10.1	0.670
LA minimum (parasternal long-axis view), mm	56 ± 15	57 ± 10	0.733
LA maximum (parasternal long-axis view), mm	73 ± 14	77 ± 12	0.147
RA minimum (four-chamber view), mm	44 ± 16	45 ± 10	0.389
RA maximum (four-chamber view), mm	63 ± 16	65 ± 13	0.377
TV diameter, mm/m^2^	24.3 ± 3.8	24.6 ± 4.1	0.812
Horizontal diameter, mm	35.6 ± 6.3	36.7 ± 6.2	0.460
Vertical diameter, mm	31.0 ± 7.9	30.5 ± 5.8	0.754
Annulus of total leaflet, mm	112 ± 25	121 ± 22	0.075
Annulus of anterior leaflet, mm	39.2 ± 8.5	36.4 ± 6.8	0.268
Annulus of posterior leaflet, mm	30.7 ± 9.1	42.4 ± 13.5	<0.001
Annulus of septal leaflet, mm	41.9 ± 9.9	42.1 ± 7.8	0.677
Total area, mm^2^	883 ± 364	914 ± 342	0.520
Anterior leaflet area, mm^2^	378 ± 207	305 ± 119	0.188
Posterior leaflet area, mm^2^	208 ± 77	327 ± 185	0.006
Septal leaflet area, mm^2^	296 ± 122	281 ± 107	0.865

Data are presented as *n* (%) or mean ± standard deviation.

LA: left atrium; LVDd: left ventricular diastolic dimension; LVDs: left ventricular systolic dimension; LVEF: left ventricular ejection fraction; PH: pulmonary hypertension (estimated right ventricular systolic pressure (RVSP) >40 mmHg); RA: right atrium; TR: tricuspid regurgitation; TRPG: transtricuspid pressure gradient; TV: tricuspid valve.

Six of 28 patients (21%) with one posterior leaflet had severe TR, whereas 11 of 23 patients (48%) with two posterior leaflets had severe TR. Patients with two posterior leaflets had a worse grade of TR before the operation (*P* = 0.046). In addition, more patients with two posterior leaflets had pulmonary hypertension (*P* = 0.034).

In patients with one posterior leaflet, the proportion of the anterior, posterior and septal leaflet was 35%, 27% and 38% of the total annular length, respectively (Fig. 4A). In patients with two posterior leaflets, the proportion of the anterior, posterior and septal leaflet accounted for 30%, 35% and 35%, respectively (Fig. 4B). The posterior leaflet area was also significantly larger and both the anterior and septal leaflet areas were smaller in patients with two posterior leaflets (posterior: *P* < 0.001, anterior: *P* < 0.001 and septal: *P* = 0.023).

Patients with two posterior leaflets had a larger tracing length and posterior leaflet area than patients with one posterior leaflet (42.4 ± 13.5 vs 30.7 ± 9.1 mm, respectively; *P* < 0.001 and 327 ± 185 vs 208 ± 77 mm^2^, respectively; *P* = 0.006).

### Comparison of parameters between patients with one versus two posterior leaflets according to tricuspid regurgitation severity

**Figure 5:**
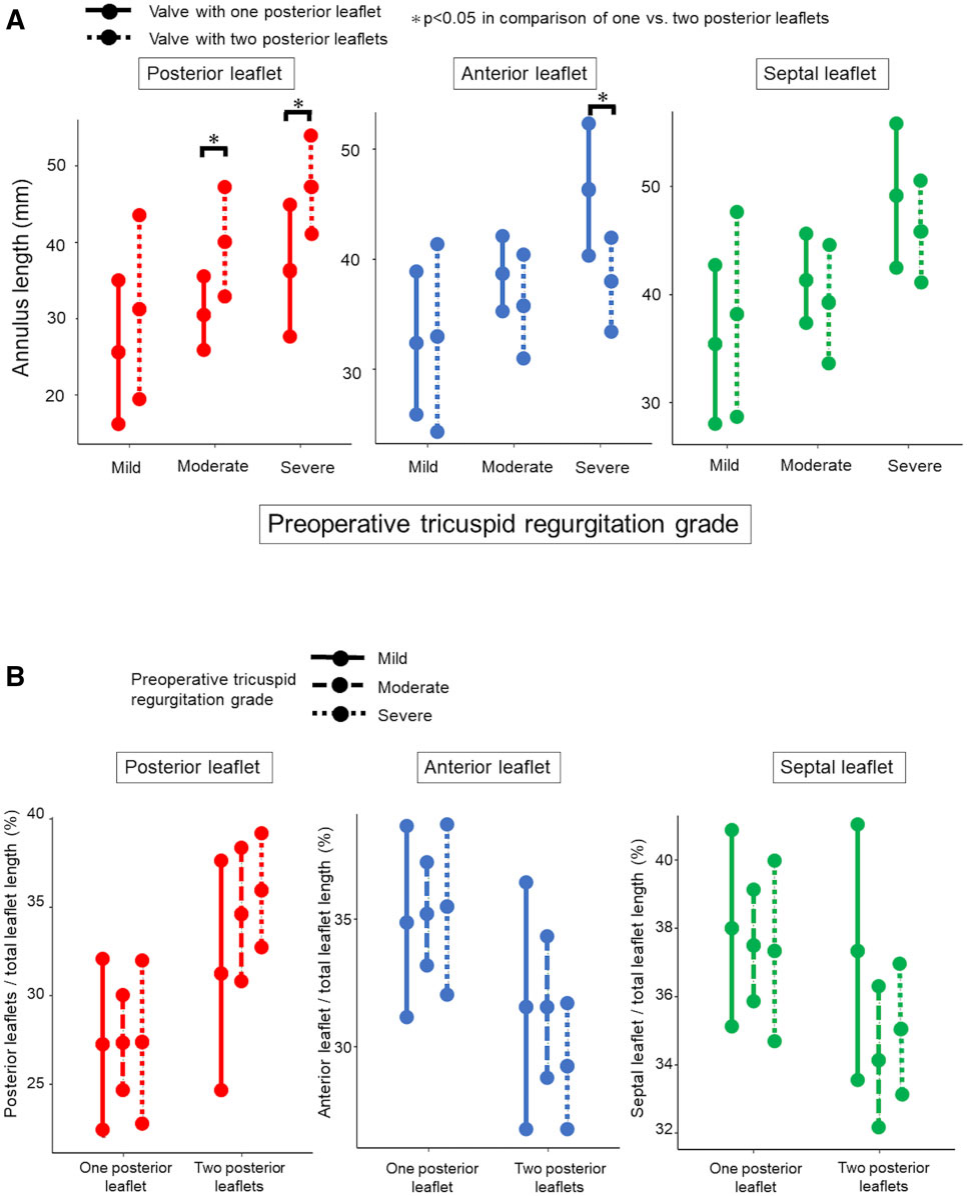
(**A**) Comparison of annulus length of each leaflet between valves with one and two posterior leaflets by tricuspid regurgitation grade. (**B**) Change in proportion of each leaflet by tricuspid regurgitation grade.

We divided the 51 patients into 3 groups according to the preoperative TR grade (≤mild, *n* = 8; moderate, *n* = 26; and severe, *n* = 17) (Fig. 5).

In the severe group, there were statistically significant differences in the annulus length of the posterior and anterior leaflets between patients with one and two posterior leaflets {posterior: 36 mm [95% confidence interval (CI), 27–45 mm] vs 48 mm (95% CI, 41–54 mm), respectively; *P* = 0.043 and anterior: 46 mm (95% CI, 40–52 mm) vs 38 mm (95% CI, 33–42 mm), respectively; *P* = 0.025}. In the moderate group, the posterior leaflet length was significantly different between patients with one and two posterior leaflets [30 mm (95% CI, 25–36 mm) vs 40 mm (95% CI, 33–47 mm), respectively; *P* = 0.038] (Fig. 5A).

Regarding the proportion of each leaflet by TR grade, there was no statistical difference between TVs with one versus two posterior leaflets. The proportion of TVs with one posterior leaflet did not change by the TR grade. However, the proportion of TVs with two posterior leaflets changed as the TR grade worsened (Fig. 5B).

## DISCUSSION

In the present study, the mid-systolic parameters of 3D TEE were correlated with the intraoperative measurements. The ratio of the posterior leaflets’ annulus and area to the whole TV in patients with two posterior leaflets was greater than that in patients with one posterior leaflet. In patients with two posterior leaflets with severe TR, the annulus length of the posterior leaflets was greater.

### Usefulness and limitation by three-dimensional transesophageal echocardiography image evaluation

The quality and resolution of computed tomography images have improved remarkably, and there are many reports of computed tomography-based evaluation of cardiac structures of the LV system, including the aortic valve or mitral valve [10]. However, compared with valves of the LV system, morphological evaluation of structures of the right ventricular system by contrast-enhanced computed tomography is difficult, and echocardiography is commonly used for preoperative evaluation. In particular, real-time 3D TEE imaging for preoperative examination of the TV morphology has attracted attention [7]. A percutaneous catheter-based procedure with an edge-to-edge clip technique for severe TR was recently established [11], and the importance of evaluating the TV morphology has been indicated. However, no software is available to measure the TV structure, and it is difficult to estimate the TV morphology preoperatively. In this study, we used the QLAB MVN/3D quantification software package to evaluate the anatomy of the TV by real-time 3D TEE imaging according to a report by Utsunomiya *et al.* [7]. Only the total length and area of the TV annulus could be measured by this software; it was difficult to measure the diameter and area of each leaflet. Therefore, we measured the perivalvular diameter and area of each leaflet by tracing the 3D echo image. It would be difficult to accurately measure the circumferential diameter of each leaflet in a plane on the 3D echo image. However, when compared with the results of the intraoperative measurements, there was no significant difference in the diameters of the leaflets in mid-systole, suggesting that this technique could be useful for preoperative evaluation. The reason for the larger difference in diastolic parameters might be that it is difficult to identify the TV commissure during diastole, and it was therefore not possible to accurately measure the respective valve leaflet diameters.

Morphological diversity of the TV, in which more than one posterior leaflet exists, has been confirmed. Sakon *et al.* [3] reported that among 100 normal autopsied hearts, 42% of TVs had two posterior leaflets and 5% of TVs had three posterior leaflets. In the present study, 29 patients (48%) had two posterior leaflets, suggesting that a similar percentage of patients with TR also had multiple posterior leaflets.

We could predict the number of posterior leaflets with 85% probability by preoperative 3D TEE imaging. The most difficult cases to evaluate the number of leaflets were those with a small RA dimension. In patients who have TR with a small RA dimension, the TV annulus is also small and the 3D structure is preserved, which may complicate image construction and judgement of the TV commissure.

### Influence of tricuspid valve morphology on regurgitation

We examined patients in whom the number of posterior leaflets was correctly assessed to determine whether there was a difference in the annulus diameter and area depending on the number of posterior leaflets. There was a significant difference in the ratio of each leaflet to the whole annulus between TVs with one and two posterior leaflets, and patients with two posterior leaflets had a larger posterior leaflet annulus length and area. Furthermore, the proportion of posterior leaflets was larger in patients with two posterior leaflets as the TR grade increased. Despite the similar annulus diameter in both patients with one and two posterior leaflets, the TR grade was more severe in patients with two posterior leaflets, which indicated that the presence of multiple posterior leaflets might cause worsening of TR. This may be due to an increase in the number of valve leaflets, which may lead to an increase in the number of valve leaflet coaptations and a change in the ratio of the sizes of the anterior, posterior and septal leaflets, in turn causing an unbalanced form of the TV.

### Surgical implications

We consider that selecting an appropriately sized annuloplasty ring and performing annuloplasty without deforming the TV annulus are important for accurate TV repair.

In this study, the number of TV leaflets was diverse and the size of each leaflet differed between TVs with one and two posterior leaflets. We also found the possibility that TVs with two posterior leaflets were easier to worse TR grade than TVs with one posterior leaflet.

**Figure 6:**
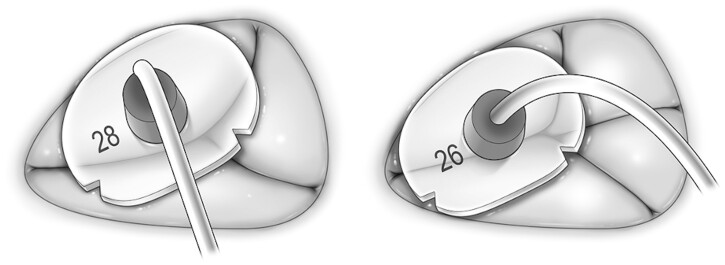
Cases of one and two posterior leaflets with the same tricuspid valve diameter. When we measured the anterior leaflet as a reference, a 28-mm annuloplasty ring was selected in valves with one posterior leaflet and a 26-mm annuloplasty ring was selected in valves with two posterior leaflets.

In the clinical setting, annuloplasty rings or bands are used without considering the characteristics of TV morphology. The measuring points when selecting a ring size differ by ring type, and the marked lines on the rings used to suture the commissure area are inconsistent by ring type. Therefore, selecting a ring size by only measuring the anterior leaflet area might underestimate the ring size, especially in patients with two posterior leaflets (Fig. 6). Figure 5A indicates that the annulus length of septal leaflet has no difference between patients with one and two posterior leaflets by the TR grade, even though there were statistically significant differences in the annulus length of the posterior and anterior leaflets between patients with one and two posterior leaflets. Annulus length of septal leaflet might be useful as a decision-making element in case of TVs with two posterior leaflets. The TV is classified as a septal leaflet and a mural leaflet. Equal plication of the mural leaflet and part of the septal leaflet is useful especially in cases of two posterior leaflets not to deform mural leaflet.

By knowing in advance that there are two leaflets, the plication method for tricuspid annulus can be determined to some extent.

In addition, a method of fitting the marked line of the ring to the anteroposterior commissure might deform the TV shape when the annuloplasty ring is used in patients who have a TV with two posterior leaflets. To prevent the deformation of the TV for TV repair, it is necessary to recognize the morphological features of the TV.

### Limitations

Our study has several limitations. First, it involved a small number of patients in a single facility. This issue should be addressed in future studies containing larger numbers of patients. Second, high-quality 3D TEE images are required to properly evaluate the number of leaflets and commissure area. For this reason, improvement of examination techniques and equipment is important. Third, the measurement method in this study was tracing the plain-view 3D image of the TV. The results were tolerated statistically, but there might have been a difference from the actual intraoperative measurements. Fourth, we did not measure the coaptation area but instead measured the leaflet area, which could be seen on the 3D image. Therefore, we should investigate the TV area using images of slices at various angles to accurately evaluate the whole leaflet size. Finally, measurement results might have differed between researchers. Our measurements were supervised by a cardiologist. Two or more researchers should perform several evaluations.

## CONCLUSION

About half of patients with TR had a TV with two posterior leaflets, and the features of such TVs were a larger annulus and area of the posterior leaflets. Preoperative 3D TEE is useful for morphological evaluation and accurate TV repair.

## Data Availability

All relevant data are within the manuscript.

## ABBREVIATIONS

CI: Confidence interval
3D: Three-dimensional
LV: Left ventricular
MVN: Mitral valve navigation
RA: Right atrial
TEE: Transoesophageal echocardiography
TR: Tricuspid regurgitation
TTE: Transthoracic echocardiography
TV: Tricuspid valve
